# Supplementary material for: HP1-Mediated Silencing of the Doublesex1 Gene for Female Determination in the Crustacean Daphnia magna
Source: J Dev Biol. 2025 Jul 3;13(3):23. doi: 10.3390/jdb13030023 (PMC12286036; doi:10.3390/jdb13030023)
Supplement: Supplementary file 1 [file jdb-13-00023-s001.zip › Supplementary Figures.pdf]

Supplementary Figures

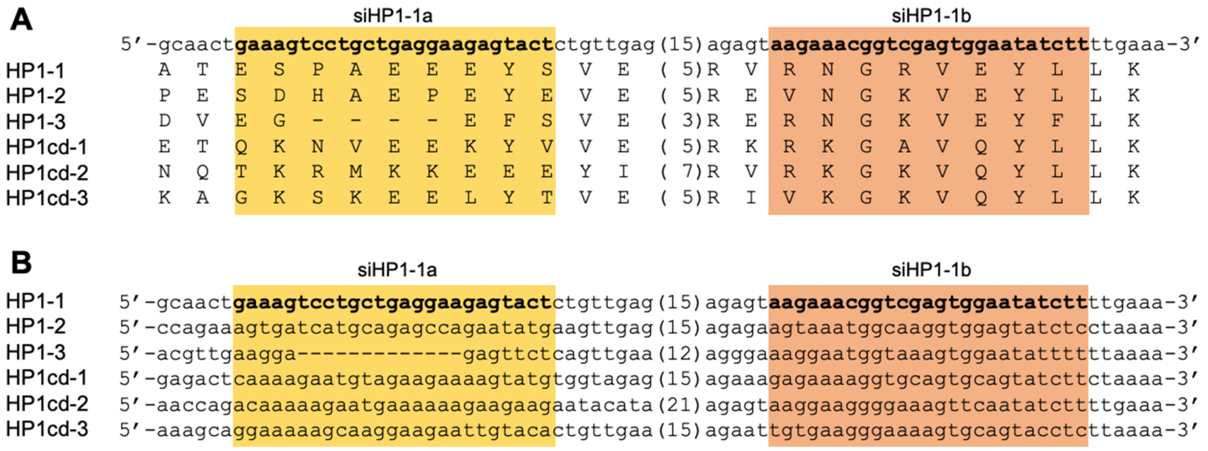

Supplementary Figure S1. Sequence alignment of HP1-1 siRNA target sites and related HP1 orthologs. (A) Amino acid sequence alignment of HP1 orthologs in *Daphnia magna* spanning the HP1-1 siRNA-a (yellow box) and siRNA-b (orange box) target regions. HP1-1, HP1-2, and HP1-3 represent full-length HP1 paralogs, while HP1cd-1, HP1cd-2, and HP1cd-3 contain only the chromodomain. Identical and mismatched residues are shown to illustrate sequence specificity of each siRNA. (B) Nucleotide sequence alignment corresponding to the regions shown in (A). Target sites of HP1-1 siRNA-a and siRNA-b are highlighted in yellow and orange, respectively. The alignment shows the extent of sequence similarity across paralogs, confirming that both siRNAs exhibit high specificity to HP1-1, with multiple mismatches relative to other HP1-related genes.

```
                                                                   Chromodomain
           HP1-1     MGRST---KGKEKEVLDEPTPATPATESPAEEEYSVEKVMDRRVR-NGRVEYLLKWKGYD
Daphnia    HP1-2     MGRSVNTTKGKKKAVQDKPSSPESDHAEP---EYEVEKVVDRREV-NGKVEYLLKWIGYG
           HP1-3     MGRNS---KVKEKEVLDEPSPGAADVEG----EFSVEKVMDRRER-NGKVEYFLKWKGFG
           HP1a      ----------MGKKIDNPESSAKVSDAEEEEEEYAVEKIIDRRVR-KGKVEYYLKWKGYP
Drosophila HP1b      -------------------------------MAEFSVERVEDKRTV-NGRTEYYLKWKGYP
           HP1c      --------------------------MVKNEPNFVVERIMDKRITSEGKVEYYIKWRGYT
           HP1alpha  --------------MGKKTKRTADSSSSEDEEEYVVEKVLDRRVV-KGQVEYLLKWKGFS
Human      HP1beta   -------------MGKKQNKKKVEEVLEEEEEEYVVEKVLDRRVV-KGKVEYLLKWKGFS
           HP1gamma  ---MASNKTTLQKMGKKQNGK-SKKVEEAEPEEFVVEKVLDRRVV-NGKVEYFLKWKGFT
                                                     :: **:: *:*   :*:.** :** *:

           HP1-1     DEDNTWEPEENLDCPALISEFEEKRKEKE--KEKKKEVEKKEKKRSLPTEDRDRKSSEEK
Daphnia    HP1-2     DDDNTWEPVEALECHELIEEFEKKRKENE--QEKKKSSESKTNKSDEIKTKKQAETKEKK
           HP1-3     EEDNTWEPEENLDCPALIAEFENARKEKE--KGKKKENEKKEKKRSLASEDSDIKAVEDK
           HP1a      ETENTWEPENNLDCQDLIQQYEASRKDEEKSAASKKDRPSSSAKAKETQGRASSSTSTAS
Drosophila HP1b      RSENTWEPVENLDCPDLIANFEESLKNNK-----------KETKKRLSTSSTPESIRSKR
           HP1c      SADNTWEPEENCDCPNLIQKFEESRAKSK-------------------------KRGEK
           HP1alpha  EEHNTWEPEKNLDCPELISEFMKKYKKMK--EGENNKPREKSESNKRKSNFSNSADDIKS
Human      HP1beta   DEDNTWEPEENLDCPDLIAEFLQSQKTAH--ETDKSE----GGKRKADSDSEDKGEESKP
           HP1gamma  DADNTWEPEENLDCPELIEAFLNSQKAGK--EKD-------GTKRKSLSDSE--SDDSKS
                       .***** :  :*  **  :       .
                                                            Chromoshadow domain
           HP1-1     RPAKKKAAEEEN-----------------RPRGFERGLDPEKIIGATD--SSGELMFLMK
Daphnia    HP1-2     RPSSNKDRDKQPMEKAHGRKEAEGVNHTSNPRGTELDQEPEVIIGATDNFHKGELAFLVK
           HP1-3     KPTKKKASECPPQDDIP------------RPKGFDRKLEPEMIIGASN--DTGELCFLMK
           HP1a      KRKSEEPTAPSGNKSKRTTDAEQDTIPVSGSTGFDRGLEAEKILGASD--NNGRLTFLIQ
Drosophila HP1b      KSFLEDDTEEQK-----------------KLIGFERGLEASKILGATD--SSGHLMFLMK
           HP1c      KPKCEEIQ---------------------KLRGYERGLELAEIVGATD--VTGDIKYLVR
           HP1alpha  KKKREQSN--------------------DIARGFERGLEPEKIIGATD--SCGDLMFLMK
Human      HP1beta   KKKKEES---------------------EKPRGFARGLEPERIIGATD--SSGELMFLMK
           HP1gamma  KKKRDAA---------------------DKPRGFARGLDPERIIGATD--SSGELMFLMK
                     :   .                           *     :   *:**::    * : :*::

           HP1-1     WKSSDEADLVPARQANSKCPQIVIQFYEERLTWHSNTNDDDGESKEKDGTD---------
Daphnia    HP1-3     WKSSDEADLVPASQANRLCPQVVIQFYEERLTWHNNTSSDEAKSKEKERVE---------
           HP1-2     WKSSDTPEFVLSSRANVMWPQMVIKFYESRLQWSSNAMEGEEIDTLE-------------
           HP1a      FKGVDQAEMVPSSVANEKIPRMVIHFYEERLSWYSDNED---------------------
Drosophila HP1b      WKGSDHADLVPAKLANTRCPQVVIQFYEERLTWHTGSGNGNGNTNSVNLGSSGGLGSVGG
           HP1c      WQFCDEFDLVPSAQIVEKDPQMLIDYFQKMAPYSRHIAMRMKGVPEELRLAASRTSYPHI
           HP1alpha  WKDTDEADLVLAKEANVKCPQIVIAFYEERLTWHAYPEDAENKEKETAKS----------
Human      HP1beta   WKNSDEADLVPAKEANVKCPQVVISFYEERLTWHSYPSEDDDKKDDKN------------
           HP1gamma  WKDSDEADLVLAKEANMKCPQIVIAFYEERLTWHSCPEDEAQ------------------
                     ::  *  ::* :       *:::* :::.   :

           HP1-1     ------------------------------------------------------------
Daphnia    HP1-3     ------------------------------------------------------------
           HP1-2     ------------------------------------------------------------
           HP1a      ------------------------------------------------------------
Drosophila HP1b      SGAGDDTAPGSVGTTGGGSNIDGGDEEDPEPASPIGSINQDENIKPDESSELDNGQPDAD
           HP1c      SSAPVEVPPEVDQSAELAGHLGGIAPQVDQAPQHHAPMDLANDTDDLASVSYSIPVPGVG
           HP1alpha  ------------------------------------------------------------
Human      HP1beta   ------------------------------------------------------------
           HP1gamma  ------------------------------------------------------------

           HP1-1     ------------
Daphnia    HP1-3     ------------
           HP1-2     ------------
           HP1a      ------------
Drosophila HP1b      D-----------
           HP1c      DIAIDVPMAENQ
           HP1alpha  ------------
Human      HP1beta   ------------
           HP1gamma  ------------
```

Supplementary Figure S2. Multiple sequence alignment of HP1 orthologs across Daphnia, Drosophila, and humans. Amino acid sequences of HP1 orthologs from *Daphnia magna* (HP1-1, HP1-2, HP1-3), *Drosophila melanogaster* (HP1a, HP1b, HP1c), and humans (HP1α, HP1β, HP1γ) were aligned to compare conserved domains. The chromodomain (CD) and chromoshadow domain (CSD) are indicated at the top. The chromodomain region is shaded in pink, highlighting the conserved aromatic cage residues involved in H3K9me3 recognition, which are marked with red circles. The chromoshadow domain is shaded in green, and residues involved in the dimerization surface are indicated with blue circles. Asterisks (*), colons (:), and periods (.) below the alignment indicate fully conserved, strongly similar, and weakly similar residues, respectively. This alignment confirms that the *Daphnia* HP1-1 protein possesses key conserved features required for histone binding and dimerization, supporting its proposed function as a chromatin-associated silencing factor analogous to canonical HP1 proteins in other metazoans.

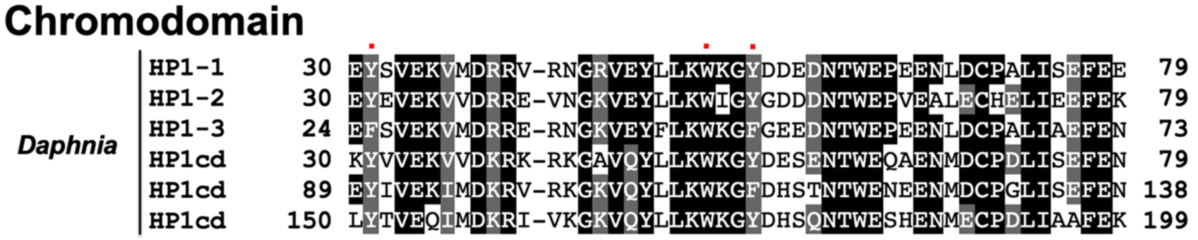

Supplementary Figure S3. Alignment of the chromodomain region in Daphnia magna HP1 homologs. Amino acid sequences of the chromodomain (CD) region from six HP1-related proteins in *Daphnia magna* (HP1-1, HP1-2, HP1-3, and three HP1cd variants) are aligned. Conserved residues that form the aromatic cage responsible for H3K9me3 recognition are indicated with red dots above the alignment. Black and gray shading denote conserved (black) or similar (gray) residues across the sequences. The alignment shows that the key residues for H3K9me3 binding are well conserved among HP1 homologs, supporting their functional conservation in heterochromatin-mediated gene silencing.

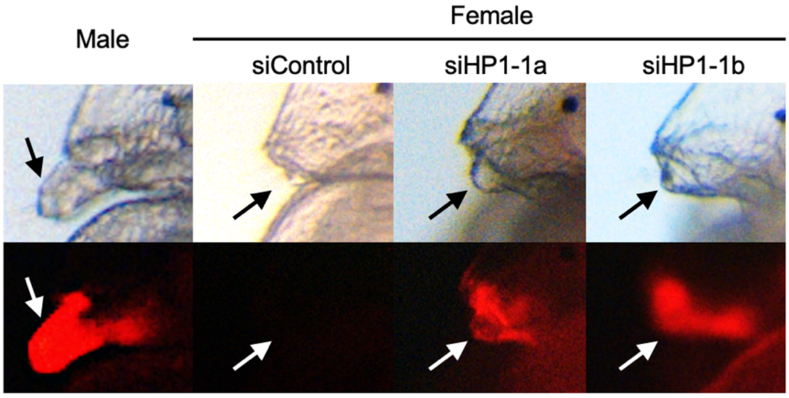

Supplementary Figure S4. Enlargement of the first antennae in HP1-1 RNAi juveniles. Bright-field (top row) and mCherry fluorescence (bottom row) images show the anterior region of male (left) and female (right three panels) juveniles derived from the Dsx1 reporter strain. Arrows indicate the first antennae. In HP1-1 knockdown females (siHP1-1a and siHP1-1b), elongation of the first antennae and upregulation of mCherry fluorescence were observed, resembling male characteristics. In contrast, siCtrl-injected females exhibited short antennae and no detectable mCherry signal.

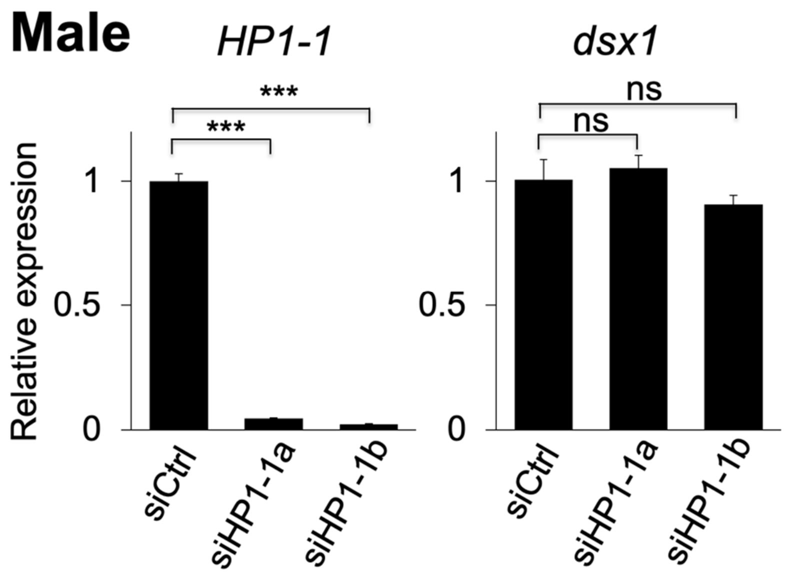

Supplementary Figure S5. Gene expression of HP1-1 RNAi males. Gene expression profiles of *HP1-1* and *dsx1* in siCtrl and siHP1-1 male embryos. RT-qPCR results were shown as expression levels normalized with the housekeeping gene *ribosomal protein L32*. Error bars indicate S.E.M (n = 3). ***p<0.001, ns: not significant (Student's T-test).
